# Factors Associated with Immunosuppressive Adherence in Liver Transplant Recipients Treated with Once- and Twice-Daily Tacrolimus

**DOI:** 10.3390/medicina62071391

**Published:** 2026-07-18

**Authors:** Sami Akbulut, Betul Ozdemir, Ummuhan Akturk, Kemal Baris Sarici

**Affiliations:** 1 Department of Surgery and Liver Transplantation Institute, Faculty of Medicine, Inonu University, Malatya 44280, Türkiye; 2 Department of Biostatistics and Medical Informatics, Faculty of Medicine, Inonu University, Malatya 44280, Türkiye; 3 Department of Public Health, Faculty of Nursing, Inonu University, Malatya 44280, Türkiye

**Keywords:** liver transplantation, immunosuppressive agents, tacrolimus, medication adherence, psychological distress, sociodemographic factors

## Abstract

*Background and Objectives*: Immunosuppressive medication adherence is essential for maintaining graft function and long-term survival after liver transplantation, yet adherence may be influenced by treatment complexity, psychosocial burden, and post-transplant medication changes. Although once-daily tacrolimus (TAC) has been proposed as a regimen-simplification strategy, the relationship between TAC dosing frequency and adherence in liver transplant recipients remains insufficiently clarified. This study aimed to evaluate the association between TAC dosing frequency and medication adherence in liver transplant (LT) recipients and to compare psychosocial characteristics, immunosuppressive treatment patterns, and post-transplant medication stability between dosing regimens. *Materials and Methods*: This cross-sectional study included 231 LT recipients followed at the Inonu University Liver Transplantation Institute. Patients were categorized according to TAC dosing regimen as once-daily (*n* = 121) or twice-daily (*n* = 110) use. Data were collected through structured telephone interviews using the validated Immunosuppressive Medication Adherence Scale (IMAS), together with demographic, clinical, psychosocial, treatment-related, and socioeconomic variables. *Results*: Most demographic and anthropometric characteristics were comparable between once-daily and twice-daily TAC groups, and mean IMAS total scores did not differ between groups (*p* = 0.785), indicating similar levels of self-reported medication adherence. In contrast, anxiety or depressive disorders were more frequent among once-daily TAC recipients (*p* = 0.045), and everolimus use was higher in this group (*p* = 0.025). Frequent post-transplant immunosuppressive medication changes were markedly more common among once-daily recipients (*p* < 0.001), and low monthly income was also more prevalent (*p* = 0.002). In subgroup comparisons, lower IMAS scores were observed in recipients experiencing frequent medication changes (*p* = 0.011) and in those who had not received medication-related education from healthcare professionals (*p* = 0.013). In univariate general linear model analyses, IMAS scores were not associated with TAC dosing regimen or sociodemographic characteristics, whereas frequency of immunosuppressive medication changes (*p* = 0.012), source of support for medication management (*p* = 0.048), and receipt of medication-related education from healthcare professionals (*p* = 0.036) were significantly associated with IMAS scores. In multivariable linear regression, frequent medication changes (*p* = 0.017), source of support for medication management (*p* = 0.028), and receipt of medication-related education (*p* = 0.019) remained independently associated with IMAS scores. *Conclusions*: TAC dosing frequency was not associated with self-reported medication adherence in LT recipients. Lower IMAS scores were associated with frequent post-transplant immunosuppressive medication changes, the need for external support in medication management, and lack of medication-related education from healthcare professionals. Compared with the twice-daily group, once-daily TAC recipients more frequently reported anxiety/depressive disorders, everolimus use, frequent medication changes, and low income, despite similar IMAS scores.

## 1. Introduction

Liver transplantation (LT) is the definitive treatment for end-stage liver disease and has shown excellent long-term outcomes, primarily due to advances in immunosuppression, surgical technique, and perioperative care [1,2]. The primary goals of LT are to improve quality of life, prolong survival, preserve graft function, and prevent complications such as rejection [3,4].

Despite advances in LT, primary graft dysfunction and rejection remain major challenges [5]. Although immunosuppressive therapies have improved substantially, acute rejection rates remain approximately 10–30%, while chronic rejection rates range between 3 and 20% in LT recipients [6,7,8]. Rodríguez-Perálvarez et al. [6], in a systematic review and critical appraisal of randomized trials in LT, reported that biopsy-proven acute cellular rejection remains frequent after LT, occurring in up to 40% of recipients and reaching 80% in series using protocol biopsies. Across randomized trials, rejection rates varied markedly according to the immunosuppressive strategy used, ranging from 5% in the tacrolimus (TAC)-based regimens evaluated by Ramirez et al. [9] to 66.7% in a reduced-dose TAC regimen with subsequent TAC weaning reported by Benítez et al. [10]. Traditionally, the success of LT has been evaluated based on patient and graft survival, and long-term outcomes after LT are strongly influenced by adherence to prescribed immunosuppressive therapy [11]. Immunosuppressive therapy consists of corticosteroids, calcineurin inhibitors (TAC, CsA), and mTOR inhibitors (everolimus, sirolimus), aiming to suppress the immune response and prevent graft rejection [12]. Strict adherence to immunosuppressive therapy is crucial after LT, as non-adherence can reduce treatment efficacy, leading to graft loss and increased mortality [13]. The introduction of effective immunosuppressive agents over the past three decades has significantly improved outcomes and long-term survival [14,15,16].

TAC, a potent calcineurin inhibitor, was approved by the Food and Drug Administration (FDA) in 1994 to prevent organ rejection in LT recipients. It remains one of the most commonly used immunosuppressive drugs in LT [17,18,19]. TAC inhibits T-lymphocyte activation and proliferation and is widely used as a first-line treatment for preventing acute rejection in LT recipients [19]. Although TAC has been proven highly effective in preventing graft rejection [20], its absorption and metabolism exhibit significant inter- and intra-patient variability [17,21,22]. TAC is available as immediate-release and prolonged-release formulations, with the former requiring twice-daily dosing and the latter allowing once-daily administration [19].

Due to its low water solubility, first-pass metabolism in the gastrointestinal tract, and the activity of P-glycoprotein efflux pumps in enterocytes, immediate-release TAC, which has an average oral bioavailability of approximately 20–25%, is characterized by limited and variable absorption [23,24]. Twice-daily TAC dosing has been associated with an increased risk of non-adherence, which may contribute to acute rejection and potential graft loss [21,22,24,25]. These pharmacokinetic and adherence-related challenges have raised the question of whether a once-daily TAC regimen, as a regimen-simplification strategy, may support better adherence than twice-daily dosing in this population. Notably, the oral bioavailability of prolonged-release TAC is estimated to be approximately 40% higher than that of the same dose of the immediate-release formulation, potentially contributing to more consistent drug exposure [26].

In the context of TAC-based immunosuppressive regimens, even minor deviations in dosing time or frequency may lead to clinically meaningful fluctuations in drug exposure, with potential implications for rejection risk and drug-related toxicity. Accordingly, adherence is particularly important in TAC-based immunosuppressive regimens [27]. In this setting, adherence extends beyond correct medication intake and encompasses regular clinical follow-up, therapeutic drug monitoring, and timely communication with healthcare providers regarding adverse effects [28]. Despite this clinical importance, non-adherence remains a common problem, affecting approximately 15–40% of LT recipients [1,29]. Importantly, adherence behavior in LT recipients is not solely determined by regimen complexity but is also influenced by psychosocial distress, health literacy, socioeconomic conditions, and the need for frequent post-transplant medication adjustments.

While immunosuppressive therapy is essential for preventing allograft rejection, long-term use can lead to significant adverse effects, including infections, metabolic disorders (diabetes, hypertension, osteoporosis, and dyslipidemia), renal dysfunction, cardiovascular disease, and malignancies [1,30,31]. Given these concerns, ensuring adherence to immunosuppressive medications is critical for long-term graft survival and LT recipient well-being in this population. However, whether TAC dosing frequency is associated with self-reported medication adherence independently of psychosocial burden and post-transplant treatment instability in LT recipients remains unclear. Accordingly, this study aimed to evaluate whether TAC dosing frequency was associated with self-reported medication adherence in LT recipients and to examine whether psychosocial characteristics and post-transplant treatment stability differed between dosing regimens in a real-world clinical setting.

## 2. Material and Methods

### 2.1. Type, Location, and Time of the Study

This cross-sectional, survey-based study included liver transplant recipients who underwent transplantation at the Inonu University Liver Transplantation Institute between March 2002 and February 2023, had at least one year of postoperative follow-up, and were reachable by phone at the time of study planning in February 2025. Recipients were categorized into two groups according to TAC dosing regimen: those receiving once-daily TAC (*n* = 121) and those receiving twice-daily TAC (*n* = 110). The primary outcome was the difference in IMAS total score between once-daily and twice-daily TAC recipients. Secondary outcomes included between-group differences in demographic, clinical, psychosocial, and treatment-related characteristics, as well as exploratory analyses of factors associated with IMAS total score.

### 2.2. Sample Size and Power Analysis

The sample size for this study was calculated using data from Cossart et al. [32], who compared two groups based on medication adherence using the Brief Illness Perception Questionnaire (Understanding Sub-Dimension). Their results showed that the mean score in the adherent group was 8.7 with a standard deviation of 1.5, while the mean score in the non-adherent group was 7.9 with a standard deviation of 2.1. Using an alpha level of 0.05 and a power of 95%, the minimum number of LT recipients required per group was calculated as 137, making a total of 274 participants. The sample size calculation was conducted using MedCalc^®^ Statistical Software version 20.015 (MedCalc Software Ltd., Ostend, Belgium; https://www.medcalc.org; 2021).

### 2.3. Participant Selection and Group Allocation

After calculation of the required sample size, 280 LT recipients (140 in each group) who were confirmed to be alive during the study period and were using either once-daily or twice-daily TAC were selected using random sampling from eligible recipients using R statistical software (version 4.4.3; R Foundation for Statistical Computing, Vienna, Austria). All selected LT recipients were contacted and invited to participate through structured telephone interviews. A total of 130 LT recipients from each group agreed to participate and completed the survey. The survey forms were reviewed by an independent researcher, and responses from 121 and 110 LT recipients in the once-daily and twice-daily TAC groups, respectively, were deemed eligible according to the study inclusion criteria.

### 2.4. Immunosuppressive Medication Adherence Scale (IMAS)

This scale is a validated and standardized self-reported measurement tool developed to assess medication adherence in LT recipients who have undergone solid organ transplantation and use immunosuppressive drugs. It was developed by Ozdemir Koken et al. [33,34]. The scale is applicable to LT recipients who have completed at least two months after LT. It consists of a total of 11 items and a single dimension. The scale uses a 5-point Likert-type rating system for the first eight items and a 2-point Likert-type rating system for the last three items. The first eight items are scored based on the following options: never, rarely, sometimes, often, and always. The last three items have yes and no options. The scale contains two positive (items 4 and 6) and nine negative (items 1, 2, 3, 5, 7, 8, 9, 10, 11) statements. In the 5-point Likert-type rating system, positive items are scored from 1 to 5, while negative items are reverse-scored on a 5-to-1 scale. For the last three items (9, 10, 11), “yes” is scored as 1 and “no” is scored as 5.

The minimum possible total score from the scale is 11, while the maximum is 55. In the present study, IMAS was administered in interviewer-applied form during structured telephone interviews. The total score is obtained by summing the scores of each item. The interpretation of the total score follows this principle: as the total score increases, medication adherence improves. A high total score indicates good adherence to medication and appropriate medication-taking behavior, whereas a low total score suggests poor adherence and failure to exhibit the expected medication-taking behavior. This scale has previously been used in our clinic to compare two different groups of liver transplant recipients. In that study, Cronbach’s alpha internal consistency coefficient was calculated as 0.755 [1].

### 2.5. Study Variables and Variable Definitions

Demographic variables included age, sex, body mass index (BMI), educational status, marital status, employment status, and monthly household income. Clinical and treatment-related variables included chronic comorbidities, hypertension, diabetes mellitus, osteoporosis, cardiovascular disease, chronic obstructive pulmonary disease, anxiety and/or depressive disorders, type of liver transplantation, everolimus use, prednisolone use, mycophenolate mofetil use, history of graft rejection, frequency of immunosuppressive medication changes after LT, source of support for medication management, receipt of medication-related education from healthcare professionals, and knowledge of immunosuppressive drug names. For the variable source of support for medication management, responses were categorized as any external support and no external support; the any external support category included support from a spouse, parent, child, or caregiver.

### 2.6. Study Protocol, Ethical Considerations, and Funding

This study was conducted in accordance with the ethical principles of the 1964 Declaration of Helsinki and its subsequent amendments and complied with all applicable institutional and national regulations governing research involving human participants. Ethical approval was obtained from the Institutional Review Board for Non-Interventional Clinical Research (Approval date: 11 March 2025; Approval number: 7301). The study received financial support from the Inonu University Scientific Research Projects Coordination Unit (Grant ID: TSA-2026-4607). The study was designed, conducted, analyzed, and reported in accordance with the STROBE (Strengthening the Reporting of Observational Studies in Epidemiology) guidelines to enhance transparency, reproducibility, and methodological rigor [35]. All participants were informed about the study, and verbal and/or written informed consent were obtained prior to participation.

### 2.7. Biostatistical Analysis

IBM SPSS Statistics for Windows, version 25.0 (IBM Corporation, Armonk, NY, USA) was used for all statistical analyses. Continuous variables were summarized as mean ± standard deviation (SD) with corresponding 95% confidence intervals (CI), while categorical variables were presented as frequencies and percentages. The normality of continuous variables was assessed using visual methods and the Shapiro–Wilk test, and homogeneity of variances was evaluated using Levene’s test. Continuous variables were compared between two groups using independent-samples *t*-tests and across more than two groups using one-way analysis of variance (ANOVA). Categorical variables were compared using Pearson’s chi-square test, continuity-corrected chi-square test, or Fisher’s exact test according to expected cell counts. For 2 × 2 tables, Fisher’s exact test was used when any expected cell count was ≤5, continuity-corrected chi-square test was used when expected cell counts were >5 and <25, and Pearson’s chi-square test was used when expected cell counts were ≥25. Effect sizes were reported to quantify the magnitude of group differences, including Cohen’s d for continuous variables compared between two groups (small = 0.2, medium = 0.5, large = 0.8) [36,37], eta-squared (η^2^) for ANOVA analyses (small = 0.01, medium = 0.06, large = 0.14) [36], and Cramér’s V for categorical variables (small = 0.1, medium = 0.3, large = 0.5) [38]. In addition, univariate analyses examining factors associated with the IMAS score were conducted using the General Linear Model (GLM) framework, with IMAS treated as a continuous dependent variable and sociodemographic, clinical, psychosocial, and treatment-related variables entered as fixed factors. Effect sizes for GLM analyses were reported as partial eta squared (partial η^2^) (small = 0.01, medium = 0.06, large = 0.14) [39]. Where omnibus comparisons involving more than two groups were statistically significant, post hoc pairwise comparisons were performed using Bonferroni correction. No multiple-testing correction was applied to the regression analyses. Hierarchical linear regression analysis was performed to evaluate the incremental explanatory contribution of predefined predictor blocks to IMAS scores. An additional adjusted multivariable linear regression model was performed to specifically evaluate whether the TAC dosing regimen was independently associated with IMAS score after adjustment for baseline variables that differed between groups. Multivariable linear regression analysis using the enter method was performed to identify independent factors associated with IMAS scores. Variables with a *p*-value < 0.10 in univariate analyses were entered into the final regression model. Regression coefficients were presented as unstandardized coefficients (B), standard errors (SE), standardized coefficients (β), 95% confidence intervals (CI), and *p*-values. Multicollinearity was assessed using tolerance and variance inflation factor (VIF) values. All statistical tests were two-sided, and a *p*-value < 0.05 was considered statistically significant.

## 3. Results

### 3.1. Baseline Characteristics of the Study Population

Table 1 presents the distribution of quantitative variables for the entire study population. The mean age of the participants was 51.8 ± 14.2 years (95% CI: 50–54). The mean height was 168.4 ± 9.3 cm (95% CI: 167–170), and the mean body weight was 75.5 ± 14.8 kg (95% CI: 74–77). The mean BMI was 26.6 ± 4.7 kg/m^2^ (95% CI: 26–27). The overall mean IMAS total score was 51.8 ± 3.9 (95% CI: 51–52), providing a comprehensive overview of the study cohort.

Table 2 summarizes the qualitative characteristics of the study population. The majority of participants were male (67.1%) and married (86.1%). Regarding educational level status, most participants had completed primary education (42.9%), followed by high school (19.0%), middle school (13.9%), university or higher education (11.3%), and no formal education (13.0%). Nearly half of the cohort was unemployed (46.3%), while 35.9% were retired and 17.7% were employed. Monthly household income levels were categorized as low, middle, and high in 43.7%, 45.9%, and 10.4% of participants, respectively. Overall, 42.9% of the study population had at least one chronic comorbidity, most commonly diabetes mellitus (25.1%) and hypertension (23.8%). Other comorbidities, including cardiovascular disease (6.9%), osteoporosis (0.9%), and chronic obstructive pulmonary disease (1.3%), were infrequent. Anxiety and/or depressive disorders were reported by 7.8% of recipients. LDLT was the predominant transplant type (90.9%). All LT recipients received TAC-based immunosuppression, with concomitant everolimus, prednisolone, and mycophenolate mofetil administered in 26.8%, 11.7%, and 5.6% of LT recipients, respectively; a history of acute or chronic graft rejection after LT was reported in 5.6% of recipients. In the post-transplant period, changes in immunosuppressive medication were reported by 36.4% of recipients. Most patients managed their medications without external support (77.1%), whereas 22.9% received external support for medication management. Regarding patient education, 67.1% of participants reported having received medication-related information from healthcare professionals, and awareness of immunosuppressive drug names was high, with 91.3% of recipients correctly identifying their medications.

### 3.2. Comparison of Once-Daily and Twice-Daily TAC Groups

Table 3 presents the comparison of quantitative variables between the twice-daily and once-daily groups. The mean age was similar between the twice-daily and once-daily groups (*p* = 0.855), with a negligible effect size (Cohen’s d = 0.024). Mean height did not differ significantly between groups (*p* = 0.170), showing a small effect size (Cohen’s d = 0.182). Mean body weight was also comparable between the twice-daily and once-daily groups (*p* = 0.797), with a negligible effect size (Cohen’s d = 0.034). Mean BMI was likewise similar between groups (*p* = 0.325), with a small effect size (Cohen’s d = 0.130). Similarly, mean IMAS total scores were nearly identical between the twice-daily and once-daily groups (*p* = 0.785), with a negligible effect size (Cohen’s d = 0.036).

**Figure 1:**
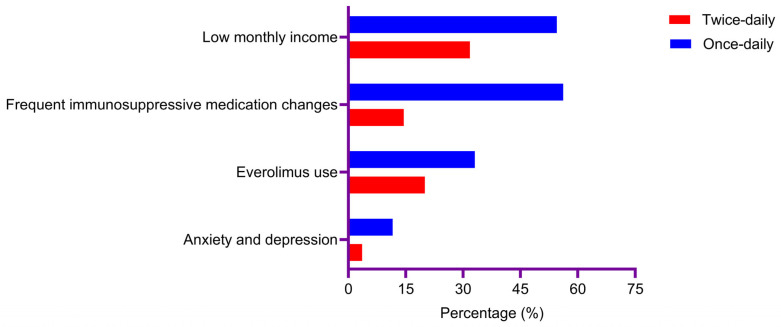
Distribution of selected significant qualitative variables in the twice-daily and once-daily TAC groups.

Table 4 presents the comparison of qualitative variables between the once-daily and twice-daily groups. Gender distribution was similar between groups (*p* = 0.612). Educational status did not differ significantly across categories (overall *p* = 0.301), nor did marital status (married: 90.0% vs. 82.6%; *p* = 0.154) or employment status (*p* = 0.981). The prevalence of chronic comorbidities was comparable (*p* = 0.761), including hypertension (*p* = 0.713), diabetes mellitus (*p* = 0.426), osteoporosis (*p* = 1.000), cardiovascular disease (*p* = 0.135), and chronic obstructive pulmonary disease (*p* = 0.606). Statistically significant differences were observed in selected variables. Anxiety or depression was more frequent in the once-daily group (11.6% vs. 3.6%; *p* = 0.045). Everolimus use was also higher among once-daily recipients (33.1% vs. 20.0%; *p* = 0.025). Post-transplant medication instability differed markedly between groups, with frequent medication changes reported by 14.5% of twice-daily recipients compared with 56.2% of once-daily recipients (*p* < 0.001; effect size = 0.432). Socioeconomic status differed significantly between groups, as low monthly income was more common in the once-daily group (54.5% vs. 31.8%; *p* = 0.002), whereas moderate and good income levels were more prevalent among twice-daily recipients. Overall, compared with the twice-daily group, the once-daily group showed higher frequencies of anxiety/depression, everolimus use, frequent medication changes, and low monthly income. The distribution of selected significant qualitative variables between once-daily and twice-daily TAC recipients is shown in Figure 1.

### 3.3. Subgroup Comparisons of IMAS Scores

Table 5 presents the comparison of mean IMAS scores between TAC dosing regimens and selected clinical subgroups. Mean IMAS total scores were similar between the twice-daily and once-daily TAC groups (*p* = 0.785), with a negligible effect size (Cohen’s d = 0.036). According to gender, mean IMAS scores did not differ significantly between male and female recipients (*p* = 0.183), showing a small effect size (Cohen’s d = 0.187). Similarly, mean IMAS scores were comparable between recipients with and without chronic comorbidities (*p* = 0.264), with a small effect size (Cohen’s d = 0.142). No significant difference in mean IMAS scores was observed between recipients with and without anxiety or depression (*p* = 0.637), with a negligible effect size (Cohen’s d = 0.116). When stratified by monthly income level, mean IMAS scores did not differ across groups (one-way ANOVA, *p* = 0.884), with a negligible effect size (η^2^ = 0.001). Recipients who reported frequent post-transplant medication changes had significantly lower mean IMAS scores compared with those without frequent changes (*p* = 0.011), with a small-to-moderate effect size (Cohen’s d = 0.349). Recipients receiving any external support for medication management had numerically lower mean IMAS scores than those with no external support; however, this difference did not reach statistical significance (*p* = 0.055), with a small effect size (Cohen’s d = 0.300). In addition, recipients who had received medication-related education from healthcare professionals demonstrated significantly higher IMAS scores than those who had not (*p* = 0.013), with a moderate effect size (Cohen’s d = 0.351).

### 3.4. Univariate Analysis of Factors Associated with Medication Adherence

Table 6 presents the results of univariate general linear model analyses evaluating factors associated with IMAS scores. The overall univariate general linear model was not statistically significant (*p* = 0.079) and explained a modest proportion of variance in IMAS scores (R^2^ = 0.105; adjusted R^2^ = 0.038)**.** The analyses demonstrated that IMAS total scores did not differ according to TAC dosing regimens, with no significant difference in adherence scores between patients receiving twice-daily and once-daily doses (*p* = 0.657; partial η^2^ = 0.001). Similarly, IMAS scores were not significantly associated with sex (*p* = 0.580; partial η^2^ = 0.001), educational level (*p* = 0.281; partial η^2^ = 0.023), marital status (*p* = 0.942; partial η^2^ = 0.001), employment status (*p* = 0.702; partial η^2^ = 0.003), monthly household income level (*p* = 0.847; partial η^2^ = 0.002), presence of chronic comorbidities (*p* = 0.439; partial η^2^ = 0.003), or the presence of anxiety and/or depressive disorders (*p* = 0.985; partial η^2^ = 0.001). Across these sociodemographic and clinical variables, effect sizes were small, indicating limited explanatory value for IMAS scores.

In contrast, several treatment-related factors showed statistically significant associations with medication adherence. IMAS scores differed significantly according to the frequency of immunosuppressive medication changes after liver transplantation, with patients who did not experience medication changes demonstrating higher adherence scores (*p* = 0.012; partial η^2^ = 0.029). IMAS scores were also significantly associated with the source of support for medication management, with lower scores observed among recipients receiving any external support than among those with no external support (*p* = 0.048; partial η^2^ = 0.018)**.** Finally, receipt of medication-related education from healthcare professionals was associated with significantly higher IMAS scores (*p* = 0.036; partial η^2^ = 0.020). Overall, these analyses suggest that among the evaluated variables, treatment stability, source of support for medication management, and receipt of medication-related education were the only variables showing statistically significant associations with IMAS scores.

### 3.5. Hierarchical Linear Regression Analysis of Predictor Blocks Associated with IMAS Scores

Table 7 shows the hierarchical linear regression analysis evaluating the incremental explanatory contribution of different predictor blocks for IMAS scores. In Model 1, the TAC dosing regimen alone explained virtually none of the variability in IMAS scores (R = 0.018, R^2^ = 0.000, adjusted R^2^ = −0.004, standard error of the estimate = 3.923), and the model was not statistically significant (F = 0.075, *p* = 0.785). In Model 2, the addition of sociodemographic variables, including age, sex, educational level, and monthly household income, increased the explained variance slightly but non-significantly (R = 0.153, R^2^ = 0.023, adjusted R^2^ = 0.002, ΔR^2^ = 0.023, F change = 1.326, *p* for ΔR^2^ = 0.261; overall model *p* = 0.374). In Model 3, adding clinical and psychosocial variables, including chronic comorbidities, anxiety and/or depressive disorders, and everolimus use, also did not significantly improve the model (R = 0.204, R^2^ = 0.041, adjusted R^2^ = 0.007, ΔR^2^ = 0.018, F change = 1.398, *p* for ΔR^2^ = 0.244; overall model *p* = 0.300). In Model 4, the final block, including receipt of medication-related education from healthcare professionals, source of support for medication management, and frequency of immunosuppressive medication changes after LT, provided a statistically significant but modest increase in explained variance (R = 0.313, R^2^ = 0.098, adjusted R^2^ = 0.053, ΔR^2^ = 0.057, F change = 4.584, *p* for ΔR^2^ = 0.004). The final hierarchical model was statistically significant overall (F = 2.166, *p* = 0.017), but its adjusted R^2^ of 0.053 indicated limited explanatory capacity.

To further address potential confounding due to baseline differences between TAC dosing groups, an additional adjusted multivariable linear regression model was performed. In this model, IMAS total score was entered as the dependent variable, TAC dosing regimen was entered as the main independent variable, and anxiety and/or depressive disorders, everolimus use, monthly household income, and frequency of immunosuppressive medication changes after LT were entered as covariates. After adjustment for these variables, the TAC dosing regimen was not independently associated with the IMAS score (B = 0.624, SE = 0.582, β = 0.080, *p* = 0.285, 95% CI: −0.524 to 1.771; VIF = 1.294).

### 3.6. Multivariable Linear Regression Analysis of Factors Associated with Medication Adherence

**Figure 2:**
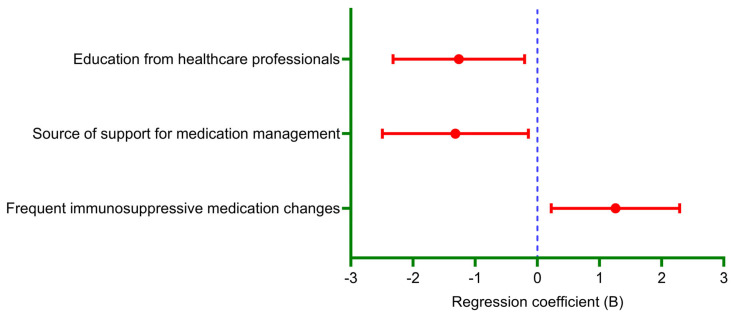
Forest plot of unstandardized regression coefficients (B) and 95% confidence intervals for factors independently associated with IMAS total score in the multivariable linear regression model.

Table 8 demonstrates that to identify independent variables associated with the total IMAS score, a multivariable linear regression analysis using the enter method was performed. Variables that showed a *p*-value < 0.10 in univariate analyses were included in the model. The total IMAS score was defined as the dependent variable, while frequency of immunosuppressive medication changes, source of support for medication management, and receipt of medication-related education from healthcare professionals were entered as independent variables. The overall regression model was statistically significant (*p* = 0.001). The model explained 6.9% of the total variance in IMAS scores (R^2^ = 0.069; adjusted R^2^ = 0.056), indicating modest explanatory capacity. According to the multivariable linear regression analysis, frequency of immunosuppressive medication changes remained independently associated with the IMAS score (B = 1.258; β = 0.155; *p* = 0.017). The direction of this association should be interpreted in light of variable coding, indicating that treatment stability was associated with higher medication adherence. Similarly, receiving any external support for medication management was independently associated with lower IMAS scores (B = −1.319; β = −0.142; *p* = 0.028)**.** In addition, receipt of medication-related education from healthcare professionals remained independently associated with the IMAS score (B = −1.264; β = −0.152; *p* = 0.019), with the negative coefficient reflecting variable coding and indicating higher IMAS scores among recipients who had received such education. No evidence of multicollinearity was detected among the variables included in the model; all VIF values were close to 1. These findings suggest that treatment-related factors and support structures were independently associated with IMAS scores, although the explanatory capacity of the model was limited. These independent associations identified in the multivariable linear regression model are visually summarized in Figure 2.

## 4. Discussion

Adherence to immunosuppressive therapy is one of the most crucial factors influencing positive clinical outcomes in LT recipients [40]. The complex and long-term nature of immunosuppressive regimens may adversely affect patient outcomes [41,42], particularly when suboptimal adherence leads to dose escalation or prolonged exposure to nephrotoxic agents. In this clinical context, post-transplant kidney failure represents a major cause of morbidity and mortality, underscoring the need for careful dose optimization to balance rejection prevention against the risk of drug-induced nephrotoxicity [43,44,45,46]. Overall, optimal immunosuppressive therapy requires a finely maintained balance to ensure long-term graft survival, minimize adverse effects, and support post-transplant quality of life in LT recipients. Accordingly, continuous monitoring of patients’ adherence to immunosuppressive medications after LT and increasing awareness of this issue remain imperative [47,48].

Immunosuppressive medication adherence in solid organ transplant recipients is influenced by multiple interrelated factors rather than by a single determinant. Factors associated with reduced adherence include longer time since transplantation, demanding or shift-based employment, treatment fatigue related to long-term polypharmacy, forgetfulness, medication burden, dosing frequency, and the perception of being fully recovered. Irregular daily routines, work-related constraints, and declining risk perception over time may further impair adherence. In contrast, structured and repeated medication education, close follow-up, active family involvement, and effective physician–patient communication appear to support adherence. Psychosocial distress, including anxiety or depression, may shape the overall post-transplant experience but does not seem to act as an independent determinant of adherence. In addition, experiential factors such as hopelessness, reduced autonomy, reluctance toward long-term medication use, drug-related side effects, and social difficulties may contribute to non-adherence, particularly when combined with regimen complexity and treatment burden. Overall, these findings suggest that immunosuppressive adherence after transplantation is dynamic and multifactorial and is best addressed through individualized follow-up strategies that integrate clinical complexity, lifestyle factors, patient education, and supportive care rather than focusing only on pharmacological regimen characteristics [49,50,51,52].

In parallel with these clinical considerations, substantial efforts have been made to optimize TAC-based immunosuppressive regimens in recent years, aiming to preserve immunosuppressive efficacy while reducing adverse effects and improving ease of use. Among the factors influencing medication adherence, psychosocial determinants have emerged as one of the most critical components. In this context, once-daily TAC formulations have been increasingly investigated in the literature, with several studies focusing on how dosing simplification influences medication adherence and on identifying patient subgroups—particularly those with psychosocial vulnerability—at higher risk of adherence-related challenges [1,29].

The present study demonstrated statistically significant differences in the prevalence of anxiety or depression between recipients receiving once-daily and twice-daily TAC. However, univariate general linear model analyses indicated that anxiety or depression was not independently associated with IMAS scores in our dataset. Consistent with this finding, a previously reported study [1] likewise found no significant association between stress or depression and IMAS scores and reported no significant differences in adherence across etiological subgroups. Collectively, these findings suggest that psychological distress may characterize clinically distinct patient subgroups, but it was not independently associated with medication adherence in the present analysis. Instead, adherence appeared to be more closely related to treatment-related and contextual factors than to psychological distress alone.

The present study also identified significant differences in everolimus use between recipients receiving once-daily and twice-daily TAC, with a higher frequency of everolimus use observed in the once-daily group. This finding may reflect greater clinical complexity and treatment individualization in this subgroup; however, the present study was not designed to evaluate the treatment indications underlying everolimus use. Accordingly, the difference in everolimus use should not be interpreted as evidence of a direct relationship between everolimus exposure and medication adherence. Consistent with this interpretation, a previously reported study [1] demonstrated that everolimus use was not independently associated with immunosuppressive medication adherence in LT recipients; similarly, in the present study, no significant difference in IMAS scores was observed between recipients receiving everolimus and those not receiving everolimus.

An alternative explanation for the greater treatment-related and psychosocial complexity observed in the once-daily TAC group may be selection bias related to clinical decision-making. In routine practice, conversion from twice-daily to once-daily TAC may not occur randomly, but may be influenced by prior clinical course, treatment instability, suspected adherence difficulties, adverse effects, or the need for regimen simplification. Therefore, the higher frequency of medication changes and anxiety or depressive disorders among once-daily TAC recipients may partly reflect the clinical history leading to conversion rather than the consequences of the once-daily regimen itself. Because the present study was cross-sectional and did not capture the timing or indication for TAC formulation conversion, these findings should be interpreted as associations rather than causal effects.

Numerous studies have suggested that a reduction in dosing frequency may contribute to improved medication adherence [53,54]. In this context, Beckebaum et al. [53] demonstrated that conversion from a twice-daily to a once-daily TAC regimen in stable LT recipients was associated with a marked reduction in immunosuppressive non-adherence, decreasing from 66.4% to 30.9%. Similarly, Bzeizi et al. [55] reviewed once-daily versus twice-daily TAC-based regimens after liver transplantation and highlighted the critical role of adherence, noting that non-adherence accounts for approximately 20% of late acute rejection episodes and 16–36% of graft losses. They further reported that non-adherence affects 15–40% of LT recipients and tends to increase over time, with regimen complexity and pill burden identified as major contributing factors. Although less frequently administered regimens have been proposed as a strategy to enhance adherence, adherence itself was not quantitatively assessed, nor were dosing regimens directly compared with respect to adherence outcomes in the available meta-analytic data.

The present study showed that the overall mean IMAS score of the study cohort was 51.8, suggesting a high level of self-reported medication adherence. When stratified by TAC dosing regimen, the mean IMAS scores were 51.8 in the twice-daily group and 51.7 in the once-daily group, with no statistically significant difference between the groups. Given that the maximum possible IMAS score is 55, these findings reflect overall high adherence levels in both groups, suggesting that medication adherence was well maintained in this closely monitored transplant population despite differences in clinical and psychosocial characteristics. In contrast, in a previously published study by Akbulut et al. [1] focusing on a more specific patient population, IMAS scores were substantially lower, a finding that may be explained by differences in patient characteristics, clinical context, and study population composition.

Beyond the present findings, IMAS has already been applied in kidney, liver, and mixed solid-organ transplant populations, although the available literature remains predominantly Türkiye-based. The original development study reported IMAS validity and an internal consistency coefficient of α = 0.610 [33]. In kidney transplant recipients, reported mean IMAS scores ranged from 43.4 ± 3.5 to 50.2 ± 3.7, and better adherence was associated with text message reminders, higher perceived social support, and more favorable health perception [56,57,58,59]; in the randomized kidney transplant study, study-specific IMAS reliability was α = 0.580 at pretest and α = 0.600 at posttest [56]. In liver transplant recipients, mean IMAS scores ranged from 47.3 ± 4.9 to 48.1 ± 6.6, while lower adherence was associated with longer time since transplantation, HCC status, frequent post-transplant medication changes, and illness perception; adherence also improved after web-based education [1,33,34,60,61]. In the Akbulut et al. [1] cohort, internal consistency coefficients were 0.755 overall, 0.694 in the HCC subgroup, and 0.816 in the non-HCC subgroup, whereas Kilinc and Yetis Demir [61] reported α = 0.74 and Dolanbay and Ozkan [60] reported α = 0.498. In a mixed solid-organ transplant cohort, Gunduz et al. [52] reported a mean IMAS score of 40.9 ± 4.1 and identified individual, regimen-related, and social-support factors as key influences on adherence. In the present study, the internal consistency of IMAS was α = 0.627 overall, with subgroup-specific coefficients of α = 0.650 in the twice-daily TAC group and α = 0.612 in the once-daily TAC group. Although these reliability coefficients were relatively modest, they were broadly comparable to those reported in several previous IMAS studies and suggest that IMAS may still provide clinically informative data, although measurement limitations should be acknowledged. Taken together, these studies suggest that IMAS has generated clinically coherent findings across different transplant settings, although broader validation in non-Türkiye populations remains limited, and further prospective studies incorporating more objective adherence measures are warranted.

Compared with IMAS, the Basel Assessment of Adherence to Immunosuppressive Medications Scale (BAASIS) has been used more extensively in international transplantation research [62]. In LT recipients, BAASIS-based studies have reported substantial non-adherence, including a 41% non-adherence rate in one cross-sectional cohort [63], while other studies showed improved adherence after conversion from twice-daily TAC to once-daily formulations, with non-adherence decreasing from 66.4% to 30.9% in one study [53], adherence increasing from 51% to 80% in another [64], and full adherence during follow-up was reported more often in the once-daily group in a pilot randomized trial [65]. Therefore, whereas our IMAS-based analysis did not show a significant adherence difference between once-daily and twice-daily TAC regimens, prior BAASIS-based liver transplant studies have detected clinically meaningful non-adherence and improvement after regimen simplification. This discrepancy may reflect not only differences in study population and clinical context but also differences in how adherence is measured by the instrument itself.

In the present study, there was no statistically significant relationship between IMAS scores and gender, chronic comorbidities, or psychological distress. In contrast, IMAS scores were significantly lower among recipients who experienced frequent post-transplant immunosuppressive medication changes and those who had not received structured medication-related education. IMAS scores also differed according to the source of support for medication management, with lower scores observed among recipients requiring any external support; however, this finding should be interpreted cautiously given the heterogeneity of support sources and the exploratory nature of subgroup comparisons. This finding suggests that frequent medication modifications may reflect a more complex clinical course that challenges sustained adherence behaviors. In contrast, Akbulut et al. [1] reported a statistically significant association between IMAS scores and both HCC status and frequent changes in immunosuppressive regimens, with higher adherence scores observed among non-HCC recipients and those experiencing frequent immunosuppressive drug modifications. The discrepancy between these findings may be attributable to differences in study populations, clinical complexity, and adherence assessment methods; an additional explanation may relate to differences in analytical approaches, as univariate general linear model analyses were not applied in the previous study, potentially limiting the assessment of independent associations between clinical factors and adherence outcomes, underscoring the need for prospective, multicenter studies to clarify the impact of treatment instability on long-term immunosuppressive adherence.

### Limitations

This study has several limitations. First, the final analyzed sample size was below the initially calculated target sample size for the primary adherence endpoint. Therefore, the absence of a statistically significant difference in IMAS scores between once-daily and twice-daily TAC recipients should be interpreted cautiously, and the possibility of a type II error cannot be excluded. Importantly, this finding should not be interpreted as evidence of equivalence or non-inferiority between the two TAC regimens. Second, data collection relied on telephone interviews rather than face-to-face assessments because regional disruption following the recent earthquake made in-person interviews impractical during the study period. Third, web-based surveys might have improved standardization, but their feasibility was limited by the relatively low literacy level of the study population. Fourth, the time since transplantation was not included as a separate analytical variable. Because medication adherence may vary over the post-transplant course, and because longer time after transplantation may be associated with treatment fatigue, lower perceived risk, medication changes, or conversion between TAC formulations, the lack of adjustment for time since LT may have introduced residual confounding and influenced the observed associations. Fifth, given the cross-sectional design and baseline differences between the two TAC groups, causality cannot be inferred and residual confounding may persist despite regression analyses. In addition, conversion to once-daily TAC in routine clinical practice may have been influenced by prior clinical course, treatment instability, suspected adherence difficulties, adverse effects, or the need for regimen simplification, which may have contributed to selection bias. Finally, the use of a self-reported adherence scale may have introduced recall and social desirability bias, and true differences in adherence between TAC dosing groups may therefore have been underestimated. Although the available IMAS literature is predominantly Türkiye-based, broader validation across different countries and transplant settings is still needed to strengthen external generalisability. Future prospective studies with larger sample sizes, adequate power for the primary adherence endpoint, longitudinal adherence assessment, and more objective adherence measures are warranted to validate these findings.

## 5. Conclusions

In this cross-sectional study of LT recipients, self-reported medication adherence did not differ between once-daily and twice-daily TAC regimens, indicating that TAC dosing frequency was not associated with IMAS scores. Although the once-daily TAC group showed greater psychosocial and treatment-related complexity, including higher rates of anxiety or depressive disorders, everolimus use, and frequent post-transplant immunosuppressive medication changes, overall adherence remained similar between groups. Lower IMAS scores were associated with frequent immunosuppressive medication changes, the need for external support for medication management, and the absence of medication-related education from healthcare professionals. These findings suggest that treatment stability, structured patient education, and supportive follow-up may be more closely associated with medication adherence than dosing frequency itself.

## Figures and Tables

**Table 1:** Quantitative characteristics of the overall study population.

Variables	Mean ± SD	95% CI
Age	51.8 ± 14.2	50–54
Height	168.4 ± 9.3	167–170
Weight	75.5 ± 14.8	74–77
BMI	26.6 ± 4.7	26–27
IMAS Total Score	51.8 ± 3.9	51–52

IMAS: Immunosuppressive Medication Adherence Scale.

**Table 2:** Qualitative characteristics of the overall study population.

Variables	Categories	Count (%)
TAC based groups	Twice-daily	110 (47.6)
	Once-daily	121 (52.4)
Sex	Male	155 (67.1)
	Female	76 (32.9)
Educational attainment	No formal education	30 (13.0)
	Primary education	99 (42.9)
	Lower secondary education	32 (13.9)
	Upper secondary education	44 (19.0)
	Tertiary education	26 (11.3)
Marital Status	Single	32 (13.9)
	Married	199 (86.1)
Employment status	Unemployed	107 (46.3)
	Employed	41 (17.7)
	Retired	83 (35.9)
Monthly household income	Low income	101 (43.7)
	Middle income	106 (45.9)
	High income	24 (10.4)
Chronic Comorbidities	Yes	99 (42.9)
	No	132 (57.1)
HT	Yes	55 (23.8)
	No	176 (76.2)
DM	Yes	58 (25.1)
	No	173 (74.9)
Osteoporosis	Yes	2 (0.9)
	No	229 (99.1)
CVD	Yes	16 (6.9)
	No	215 (93.1)
COPD	Yes	3 (1.3)
	No	228 (98.7)
Anxiety and/or Depressive Disorders	Yes	18 (7.8)
	No	213 (92.2)
Type of LT	DDLT	21 (9.1)
	LDLT	210 (90.9)
Everolimus Use	Yes	62 (26.8)
	No	169 (73.2)
Prednisolone Use	Yes	27 (11.7)
	No	204 (88.3)
Mycophenolate Mofetil Use	Yes	13 (5.6)
	No	218 (94.4)
History of Acute or Chronic Graft Rejection After LT	Yes	13 (5.6)
	No	218 (94.4)
Frequency of Immunosuppressive Medication Changes After LT	Yes	84 (36.4)
	No	147 (63.6)
Source of Support for Medication Management	Any external support	53 (22.9)
	No external support	178 (77.1)
Receipt of Medication-Related Education from Healthcare Professionals	Yes	155 (67.1)
	No	76 (32.9)
Knowledge of Immunosuppressive Drug Names	Yes	211 (91.3)
	No	20 (8.7)

Any external support: Spouse, caregiver, children, or parents.

**Table 3:** Comparison of quantitative variables between twice-daily and once-daily TAC groups.

Variables	Twice-Daily (*n* = 110)	Once-Daily (*n* = 121)	ES	*p*
Age (yr)	51.6 ± 13.6 (49–54)	51.9 ± 14.7 (49–55)	0.024	0.855
Height (cm)	167± 10 (166–169)	169 ± 8 (168–171)	0.182	0.170
Weight (kg)	75.8 ± 14.6 (73–79)	75.3 ± 15.0 (73–78)	0.034	0.797
BMI (kg/m^2^)	26.9 ± 4.2 (26–28)	26.3 ± 5.0 (25–27)	0.130	0.325
IMAS Score	51.8 ± 3.9 (51–53)	51.7 ± 4.0 (51–52)	0.036	0.785

Effect sizes are reported as Cohen’s d; IMAS: Immunosuppressive Medication Adherence Scale.

**Table 4:** Comparison of qualitative variables between twice-daily and once-daily TAC groups.

Variables (%)	Categories	Twice-Daily	Once-Daily	ES	*p*
Sex	Male	72 (65.5)	83 (68.6)	0.033	0.612
	Female	38 (34.5)	38 (31.4)		
Educational attainment	No formal educ	16 (14.5)	14 (11.6)	0.145	0.301
	Primary educ	45 (40.9)	54 (44.6)		
	Lower secondary educ	20 (18.2)	12 (9.9)		
	Upper secondary educ	17 (15.5)	27 (22.3)		
	Tertiary educ	12 (10.9)	14 (11.6)		
Marital Status	Single	11 (10.0)	21 (17.4)	0.106	0.154
	Married	99 (90.0)	100 (82.6)		
Employment status	Unemployed	51 (46.4)	56 (46.3)	0.013	0.981
	Employed	19 (17.3)	22 (18.2)		
	Retired	40 (36.4)	43 (35.5)		
Monthly household income	Low income	35 (31.8)	66 (54.5)	0.235	0.002
	Middle income	63 (57.3)	43 (35.5)		
	High income	12 (10.9)	12 (9.9)		
Chronic Comorbidities	Yes	46 (41.8)	53 (43.8)	0.020	0.761
	No	64 (58.2)	68 (56.2)		
HT	Yes	25 (22.7)	30 (24.8)	0.024	0.713
	No	85 (77.3)	91 (75.2)		
DM	Yes	25 (22.7)	33 (27.3)	0.052	0.426
	No	85 (77.3)	88 (72.7)		
Osteoporosis	Yes	1 (0.9)	1 (0.8)	0.004	1.000
	No	109 (99.1)	120 (99.2)		
CVD	Yes	11 (10.0)	5 (4.1)	0.115	0.135
	No	99 (90.0)	116 (95.9)		
COPD	Yes	2 (1.8)	1 (0.8)	0.044	0.606
	No	108 (98.2)	120 (99.2)		
Anxiety and/or Depressive Disorders	Yes	4 (3.6)	14 (11.6)	0.148	0.045
	No	106 (96.4)	107 (88.4)		
Type of LT	DDLT	10 (9.1)	11 (9.1)	0.000	1.000
	LDLT	100 (90.9)	110 (90.9)		
Everolimus Use	Yes	22 (20.0)	40 (33.1)	0.147	0.025
	No	88 (80.0)	81 (66.9)		
Prednisolone Use	Yes	16 (14.5)	11 (9.1)	0.085	0.279
	No	94 (85.5)	110 (90.9)		
Mycophenolate Mofetil Use	Yes	8 (7.3)	5 (4.1)	0.068	0.454
	No	102 (92.7)	116 (95.9)		
History of Acute or Chronic Graft Rejection After LT	Yes	4 (3.6)	9 (7.4)	0.082	0.334
	No	106 (96.4)	112 (92.6)		
Frequency of Immunosuppressive Medication Changes After LT	Yes	16 (14.5)	68 (56.2)	0.432	<0.001
	No	94 (85.5)	53 (43.8)		
Source of Support for Medication Management	Any external support	25 (22.7)	28 (23.1)	0.005	0.941
	No external support	85 (77.3)	93 (76.9)		
Receipt of Medication-Related Education from Healthcare Professionals	Yes	70 (63.6)	85 (70.2)	0.070	0.285
	No	40 (36.4)	36 (29.8)		
Knowledge of Immunosuppressive Drug Names	Yes	98 (89.1)	113 (93.4)	0.076	0.355
	No	12 (10.9)	8 (6.6)		

Effect sizes were expressed as Phi (φ) for 2 × 2 tables and Cramer’s V for tables with more than two categories; Any external support: Spouse, caregiver, children, or parents.

**Table 5:** Comparison of IMAS total scores across selected subgroups.

Independent Variables	Categories	IMAS Score	ES	*p*
TAC regimen (Groups)	Twice-daily	51.8 ± 3.9 (51–53)	0.036	0.785
	Once-daily	51.7 ± 4.0 (51–52)		
Sex	Male	52.0 ± 3.7 (51–53)	0.187	0.183
	Female	51.3 ± 4.4 (50–52)		
Educational attainment	No formal educ	51.3 ± 3.9 (50–53)	0.020	0.304
	Primary educ	52.1 ± 3.6 (51–53)		
	Lower secondary educ	50.7 ± 5.5 (49–53)		
	Upper secondary educ	51.5 ± 3.8 (50–53)		
	Tertiary educ	52.5 ± 2.7 (51–54)		
Marital status	Single	51.4 ± 3.6 (50–53)	0.105	0.590
	Married	51.8 ± 4.0 (51–52)		
Employment status	Unemployed	51.3 ± 4.4 (50–52)	0.010	0.307
	Employed	52.1 ± 3.6 (51–53)		
	Retired	52.1 ± 3.4 (51–53)		
Monthly household income	Low income	51.7 ± 3.8 (51–52)	0.001	0.884
	Middle income	51.7 ± 4.1 (51–52)		
	High income	52.1 ± 3.7 (51–54)		
Chronic Comorbidities	Yes	52.1 ± 3.1 (51–53)	0.142	0.264
	No	51.5 ± 4.4 (51–52)		
Anxiety and/or Depressive Disorders	Yes	51.3 ± 4.5 (49–54)	0.116	0.637
	No	51.8 ± 3.9 (51–52)		
Frequency of Immunosuppressive Medication Changes After LT	Yes	50.9 ± 4.2 (50–52)	0.349	0.011
	No	52.2 ± 3.7 (52–53)		
Source of Support for Medication Management	Any external support	50.9 ± 4.4 (50–52)	0.300	0.055
	No external support	52.0 ± 3.7 (51–53)		
Receipt of Medication-Related Education from Healthcare Professionals	Yes	52.2 ± 4.0 (52–53)	0.351	0.013
	No	50.8 ± 3.7 (50–52)		

Effect sizes were reported as Cohen’s d for comparisons involving two groups and as eta squared (η^2^) for comparisons involving three or more groups.

**Table 6:** Univariate GLM analysis of factors associated with IMAS total score.

Independent Variables	Categories	IMAS Score	df	F	*p*	Partial η^2^
TAC regimen (Groups)	Twice-daily	51.8 ± 3.9	1	0.198	0.657	0.001
	Once-daily	51.7 ± 4.0				
Sex	Male	52.0 ± 3.7	1	0.307	0.580	0.001
	Female	51.3 ± 4.4				
Educational attainment	No formal educ	51.3 ± 3.9	4	1.274	0.281	0.023
	Primary educ	52.1 ± 3.6				
	Lower secondary educ	50.7 ± 5.5				
	Upper secondary educ	51.5 ± 3.8				
	Tertiary educ	52.5 ± 2.7				
Marital Status	Married	51.8 ± 4.0	1	0.005	0.942	0.001
	Single	51.4 ± 3.6				
Employment status	Employed	52.1 ± 3.6	2	0.354	0.702	0.003
	Unemployed	51.3 ± 4.4				
	Retired	52.1 ± 3.4				
Monthly household income	Low income	51.7 ± 3.8	2	0.166	0.847	0.002
	Middle income	51.7 ± 4.1				
	High income	52.1 ± 3.7				
Chronic Comorbidities	Yes	52.1 ± 3.1	1	0.602	0.439	0.003
	No	51.5 ± 4.4				
Anxiety and/or Depressive Disorders	Yes	51.3 ± 4.5	1	0.000	0.985	0.001
	No	51.8 ± 3.9				
Frequency of Immunosuppressive Medication Changes After LT	Yes	50.9 ± 4.2	1	6.347	0.012	0.029
	No	52.2 ± 3.7				
Source of Support for Medication Management	Any external support	50.9 ± 4.4	1	3.948	0.048	0.018
	No external support	52.0 ± 3.7				
Receipt of Medication-Related Education from Healthcare Professionals	Yes	52.2 ± 4.0	1	4.467	0.036	0.020
	No	50.8 ± 3.7				

IMAS: Immunosuppressive Medication Adherence Scale; LT: liver transplantation. Any external support included support from a spouse, parent, child, or caregiver. Effect sizes for GLM analyses were reported as partial eta squared.

**Table 7:** Hierarchical linear regression analysis of predictor blocks associated with IMAS total score.

Model	Predictor Blocks Entered	R^2^	Adjusted R^2^	ΔR^2^	*p* for ΔR^2^	Overall Model *p*
Model 1	TAC regimen	0.000	−0.004	0.000	0.785	0.785
Model 2	Model 1 + Sociodemographic block	0.023	0.002	0.023	0.261	0.374
Model 3	Model 2 + Clinical/psychosocial block	0.041	0.007	0.018	0.244	0.300
Model 4	Model 3 + Treatment-related block and immunosuppressive medication changes	0.098	0.053	0.057	0.004	0.017

Model 1 included TAC dosing regimen. Model 2 added age, sex, educational level, and monthly household income. Model 3 additionally included chronic comorbidities, anxiety and/or depressive disorders, and everolimus use. Model 4 additionally included receipt of medication-related education from healthcare professionals, source of support for medication management, and frequency of immunosuppressive medication changes after LT. Source of support for medication management was analyzed as a binary variable: no external support versus any external support. ΔR^2^ indicates the incremental change in explained variance after adding each predictor block.

**Table 8:** Multivariable linear regression analysis of factors associated with IMAS total score.

Independent Variables	B	SE	β	*p*	95% CI	VIF
Frequency of immunosuppressive medication changes after LT	1.258	0.525	0.155	0.017	0.224 to 2.291	1.017
Source of support for medication management	−1.319	0.597	−0.142	0.028	−2.494 to −0.143	1.005
Receipt of medication-related education from healthcare professionals	−1.264	0.537	−0.152	0.019	−2.322 to −0.205	1.017
Constant	51.671	0.486	—	<0.001	50.713 to 52.630	—

B represents the unstandardized regression coefficient, indicating the expected change in IMAS score, whereas β represents the standardized regression coefficient. Source of support for medication management was analyzed as a binary variable: no external support versus any external support. Variables were coded as follows: frequency of immunosuppressive medication changes after LT, 0 = yes and 1 = no; source of support for medication management, 0 = no external support and 1 = any external support; receipt of medication-related education from healthcare professionals, 0 = yes and 1 = no. No evidence of multicollinearity was detected among the variables included in the model.

## Data Availability

The data presented in this study are available on request from the corresponding author due to privacy and ethical restrictions.
